# Modified radial tongue-shaped flap following stepwise surgical release for Benson type I camptodactyly of the 5th digit

**DOI:** 10.1038/s41598-023-31138-1

**Published:** 2023-03-09

**Authors:** Wei Liao, Lanlan Wang, Yuping Tang, Li Jiang, Ruoyi Guo, Hanjie Zhuang, Kai Tang, Pengfei Zheng

**Affiliations:** 1 Department of Orthopaedic Surgery, Children’s Hospital of Nanjing Medical University, Nanjing, 210008 China; 2 Affiliated Hospital of Integrated Traditional Chinese and Western Medicine, Nanjing University of Chinese Medicine, Nanjing, 210008 China

**Keywords:** Paediatric research, Orthopaedics

## Abstract

To investigate the outcomes of the modified radial tongue-shaped flap following stepwise surgery release for treating Benson type I camptodactyly of the 5th digit. A retrospective analysis involving patients with Benson type I camptodactyly of the 5th digit was performed. A total of 8 patients with 12 affected digits were included. Extent of surgical release depended on the degree of soft tissue contracture. Skin release, subcutaneous fascial release, and flexor digitorum superficialis tenotomy were performed in all 12 digits, sliding volar plate release in 2 digits, and intrinsic tendon transfer in 1 digit. The mean total passive motion of proximal interphalangeal joint significantly increased from 32.5° ± 16° to 86.3° ± 20.4°, while mean total active motion significantly increased from 22° ± 10.5° to 73.8° ± 27.5° (*P* < 0.05). Treatment outcomes were excellent in 6 patients, good in 3, moderate in 2, and poor in 1. Scar hyperplasia occurred in 1 patient. The radial tongue-shaped flap allowed for full coverage of the volar skin defect, and was considered aesthetically favorable. In addition, the stepwise surgical approach not only achieved good curative effects, but also allowed for individualization of treatment.

## Introduction

Camptodactyly represents a flexion deformity of the proximal interphalangeal (PIP) joint. Congenital camptodactyly is relatively common. With incidence ranging between 1 and 2%, it accounts for approximately 5% of all congenital hand deformities^1^. It is mostly autosomal dominant in inheritance.

At present, the disease is commonly classified using the classification system proposed by Benson et al.^2^, whereby type I (the most common type) appears in childhood, usually involves the 5th digit, and does not show sex predominance; type II appears during puberty and is more common among women; and type III appears at birth, usually affects several digits, and is associated with various syndromes. Severity can be divided into mild (< 30°), moderate (30°–60°), and severe (> 60°)^3^, with surgical treatment indicated for moderate-to-severe cases, and involves the release of volar contractures. However, the proper management of the volar skin defect remains a clinical issue in terms of maintaining the functionality and aesthetics of the digit. We thereby designed a radial tongue-shaped flap for coverage of the volar skin defect, and aimed to evaluate the outcomes of both the modified flap and the stepwise surgical release approach for the management of type I camptodactyly.

## Results

The mean follow-up period was 17.8 months (range 11–23 months). Skin release, subcutaneous fascial release, and flexor digitorum superficialis tenotomy were performed in all 12 digits (100%), while lumbrical muscle release was done in 2 digits (16.7%), sliding volar plate release in 2 digits (16.7%), and intrinsic tendon transfer in 1 digit (8.3%) for correction of severe PIP extension lag. At baseline, the mean total passive motion (TPM) and total active motion (TAM) of the PIP joint was 32.5° and 22°, respectively, which significantly increased to 86.3° and 73.8°, respectively (*P* < 0.05).

**Figure 1:**
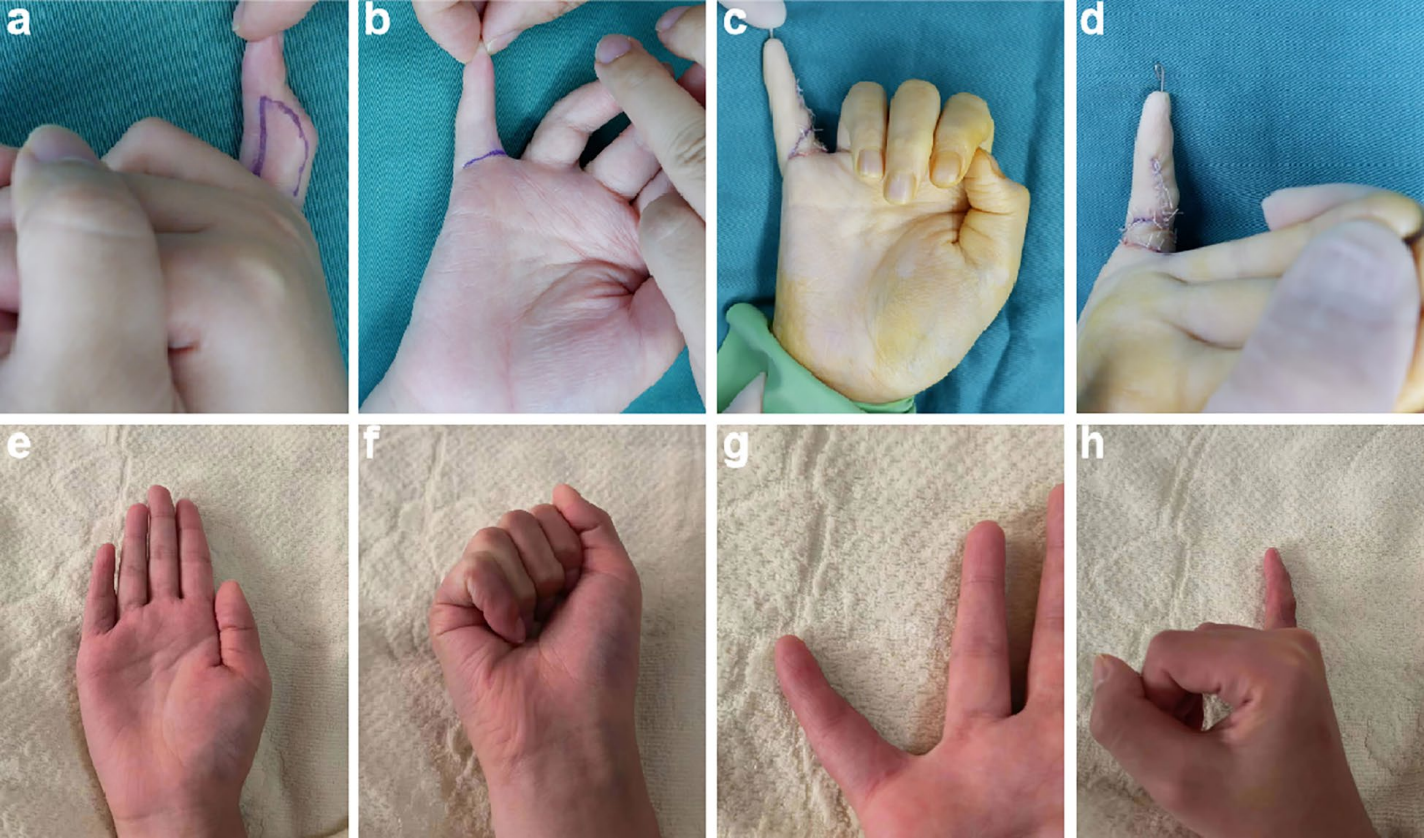
Representative images of camptodactyly seen in an 11-year-old patient. (**a**) The preoperative design of flap: the flap is designed on the radial wall of the little finger. (**b**) The palmar skin incision. (**c**) The flap was rotated 90° to completely cover the palmar skin defect. (**d**) Suture the surplus skin on the dorsal side of finger to the palmar side of skin. (**e**) The extension of the proximal interphalangeal (PIP) joint 6 months post-operation. (**f**) The flexion of the PIP joint 6 months post-operation. (**g**) The surgical incision appeared natural and had no visible scar. (**h**) The incision on the radial wall of finger showed good healing.

Treatment outcomes were excellent in 6 patients (75%), good in 3 (37.5%), moderate in 2 (25%), and poor in 1 (12.5%). All flaps survived well and remained soft in texture, and satisfactory recovery was observed in both the recipient and donor areas. No complications such as hyperpigmentation, neurovascular compromise, wound infection, or flap necrosis were observed. Scar hyperplasia was only seen in 1 patient. The results of a typical case are shown in Fig. 1, and the relevant clinical data of all patients are summarized in Table 1.

**Table 1:** Clinical characteristics of the included patients.

Serial number	Side of affected digit	Sex	Age	Preoperative TPM/TAM	Postoperative TPM/TAM	Surgical release	Skin graft	Complications	Follow-up period (months)	Treatment outcomes^6^
1	L	F	5	30/20	90/70	abce	Y	N	16	Good
2	L	M	4	35/20	60/45	abcd	N	N	17	Fair
	R			45/30	100/85	abcd	N	N	17	Good
3	L	M	11	60/40	100/100	abc	N	N	11	Excellent
	R			60/40	100/100	abc	N	N	11	Excellent
4	L	M	1	15/10	100/90	abc	N	N	20	Excellent
	R			20/15	100/90	abc	N	N	20	Excellent
5	L	M	5	20/15	40/25	abc	N	N	23	Poor
	R			20/15	100/95	abc	N	N	23	Excellent
6	L	M	1	15/10	100/100	abcef	N	N	18	Excellent
7	R	M	11	40/30	75/45	abc	N	Y	18	Good
8	L	M	1	30/20	60/40	abc	Y	N	19	Fair
Mean				32.5/22	85.4/73.8				17.8	

L, Left; R, Right; F, Female; M, Male; TPM, Total passive motion; TAM, Total active motion; a, Skin release; b, Subcutaneous fascial release; c, Flexor digitorum superficialis release; d, Lumbrical muscle release; e, Volar plate release; f: Intrinsic tendon transfer; Y, Yes; N, No.

## Discussion

Anterior–posterior flexion deformities of the PIP joint of the 5th digit was first described by Tamplin RW in 1846^4^, while the concept of camptodactyly was established by Landouzy L in 1906^5^. At present, conservative treatment is generally opted for in mild cases, especially in infants aged < 1 year. Early initiation of stretching exercises and extension support have been shown to achieve a certain degree of improvement^6^. For children aged > 1 year newly diagnosed with moderate-to-severe camptodactyly, or those who develop moderate-to-severe deformities despite conservative treatment, soft tissue surgical repair should be performed before bone maturity to allow for gradual reshaping of bones, and to avoid secondary bone and joint deformities^3^.

The pathogenesis of congenital camptodactyly can be highly varied, ranging from skin contracture, to abnormal subcutaneous fibrous tissue, abnormal flexor digitorum superficialis tendon, abnormal lumbrical muscle insertion^7^, volar plate contracture, and underdevelopment of the extensor apparatus. The timing and approach of treatment should therefore be individualized in accordance with the extent of soft tissues involved^7^.

At present, the treatment strategies and associated outcomes of 5th digit camptodactyly are highly heterogenous. The study by Evans et al.^8^ involved 31 digits in 22 patients (7 of which were type I, 8 were type II, and 7 were type III), and found no significant improvement in total range of motion of the PIP joint following surgical treatment, with improvement only observed in the extension arc of the digit. However, in the studies by Netscher and Hamilton et al. involving 18 patients with type I or II camptodactyly^9^, satisfactory results were achieved with a stepwise surgical release strategy, with 15 patients (83.3%) achieving full active range of motion of the PIP joint. With this approach, successful release can be determined based on passive flexion and extension of the digit during the operation itself. Moreover, this approach allows for the individualization of treatment, with the extent of surgical release dependent on the degree of soft tissue contracture involved, as observed in our study as well.

Among all of our patients, 8 achieved complete passive PIP joint extension upon release of the flexor digitorum superficialis. Furthermore, In this group, 5 digits obtained excellent postoperative results through the previous 3 steps of release. We therefore believe that flexion contracture deformities occurring in the skin, subcutaneous fascia, and superficial flexor digitorum muscle can achieve excellent results following thorough surgical release and good skin coverage. In addition, given that 2 of our patients aged > 10 years reported excellent and good outcomes, respectively, despite X-ray findings of secondary bone and joint changes at baseline, we believe that age does not play a role in treatment efficacy.

Volar plate release was performed in 2 patients, with treatment outcomes being excellent and good, respectively. Based on this, we considered volar plate contractures as an important pathogenesis of camptodactyly, and recommend the need for surgery, followed by an immobilization period of at least 3 weeks.

Subsequent closure of the volar skin defect also plays a significant role in the final outcome of surgery for camptodactyly. The restoration of well-functioning, natural-looking digits have long been the main subject of interest. The 2 primary considerations include (1) minimizing the length of surgical incision in the flexor tendon passage to reduce the risk of scarring and adhesions, and (2) designing more concealed flaps to reduce the visibility of scars. It is well known that skin on the dorsal side of the digit is relatively surplus; as such, attempts have been made to utilize the dorsal skin for coverage of volar skin defects. Morimoto et al.^10^ utilized a digital artery perforator flap; however, the procedure can be considered complex and extensive, and resulted in a prominent scar. Wall et al.^11^ utilized a local lateral flap, but required additional skin grafts, which not only compromised the aesthetics of the digit, but also associated with the risk of scarring of the flexor tendons. Wang et al.^12^ utilized an ulnar tongue-shaped flap, but resulted in not only a relatively prominent scar, but also one that is more likely to be exposed to rubbing, which can cause discomfort and paresthesia. As such, we instead designed a radial tongue-shaped flap, which was found to enable complete coverage of the volar skin defect. Digits flexion tendon does not need skin grafting to cover the volar wound, which can better avoid the limitation of digits flexion and extension caused by skin grafting adhesion. In this study, the good functional effect was attributed to the design and implementation of the modified flap.

Furthermore, with the flap donor site being on the radial surface, adequate coverage can be achieved with excess dorsal skin, while allowing scars to remain hidden. During surgery, we recommend that flaps should be created within the limits of the radial wall of the digit, to avoid any unnecessary incisions made on the volar surface, which can lead to scarring and contractures. Direct suturing can also be performed for closure of the donor site. Free skin grafting should be indicated in the presence of excessive flap tension, to avoid poor blood supply or scar hyperplasia, as seen in 1 of our patients. As shown in Fig. 1g, the fingers of patients in this group have a very natural appearance, with only linear scars left on the volar side. In one case, there was a slight scar hyperplasia on the lateral digital wall due to excessive suture tension. Careful operation was need to avoid the generation of scar hyperplasia.

Our modified radial tongue-shaped flap presents with several advantages. Firstly, the operation is simple, short, and relatively less invasive. In terms of postoperative outcomes, blood supply to the flap is reliable, resulting in a high flap survival rate. No flap contracture was found. Moreover, due to the presence of adipose tissue, the texture and color of skin flaps are similar to those of the recipient site. Skin flaps are thus considered superior in terms of functionality, appearance, and the lack of bloating. The recovery of sensation in the flap was rapid, given that nerve supplies are preserved. The skin flap also enables the complete coverage of volar skin defects without the risk of adhesions that can affect the flexion and extension of the digit. Finally, given the radial location of the donor flap area, scarring is hidden and not exposed to friction, and there is no concerns of contraction even if skin graft repair were needed.

However, this method has several disadvantages. Since the skin flap is supplied by the perforating branches of the proper palmar digital arteries, its resistance to tension is poor, which may necessitate skin grafting on side wall to avoid high tension suturing, as in 2 of our patients.

This study was limited by the small sample size and the short follow-up period. Large-sample studies with longer follow-ups are thus warranted. Furthermore, the Mayo clinic scoring system was used to assess postoperative outcomes^13^, which does not take into account aspects such as aesthetics.

Nevertheless, we conclude that stepwise surgical release allows for the individualization of treatment for camptodactyly, and associated with good treatment outcomes. More importantly, we found that the radial tongue-shaped flap can achieve favorable results in terms of simplicity, aesthetics and functionality, and plays a potential role in the treatment of this condition.

## Materials and methods

This study was performed in accordance with the Declaration of Helsinki and was approved by the Ethics Committee of Children’s Hospital of Nanjing Medical University (Approval number: 202205087-1). The informed consent was obtained from the parents/guardians of all participants. Patients with Benson type 1 camptodactyly treated in our institute between January 2019 and January 2021 were retrospectively analyzed. The inclusion criteria involved (1) 5th digit flexion deformity present at birth, (2) age > 1 year, and (3) moderate-to-severe deformity. The exclusion criteria involved (1) Benson types II and III camptodactyly, (2) camptodactyly in association with a syndrome, and (3) acquired camptodactyly.

A total of 12 digits (5 on the right and 7 on the left) in 8 patients (7 male and 1 female) were involved. The mean age was 4.9 years (range 1–11 years). Among them, 3 digits were previously treated conservatively. Camptodactyly was determined using the Bouvier maneuver, which involved extending the PIP joint while flexing the metacarpophalangeal joint to assess for contracture of the flexor digitorum superficialis tendon. The mean total passive motion (TPM) was 32.5°. And total active motion (TAM) of the PIP joint was 22°.

### Surgical technique

**Figure 2:**
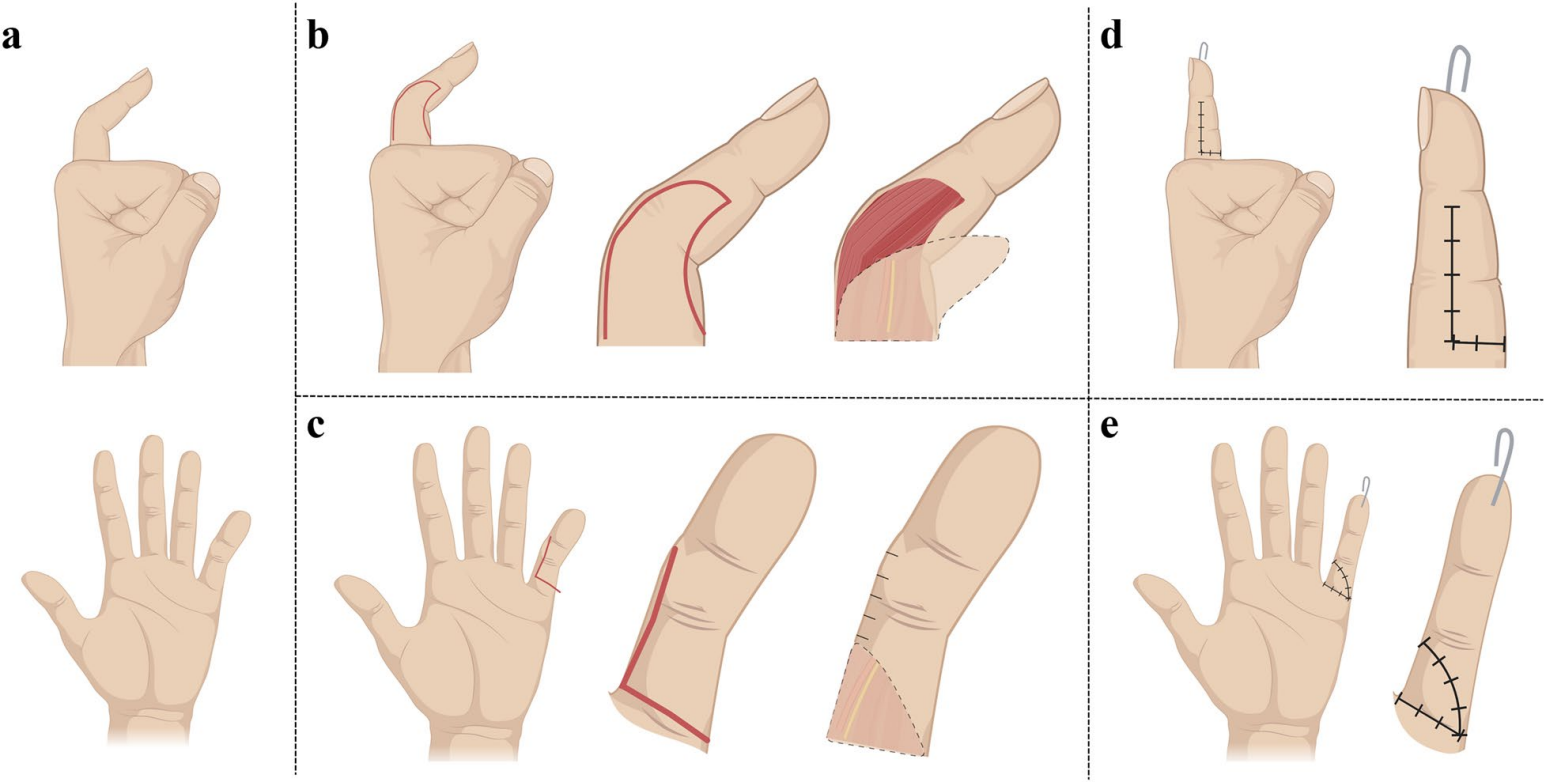
Representative illustrations of camptodactyly of the 5th digit. (**a**) Frontal and lateral views of the 5th digit before surgery. (**b**) Design of the tongue-shaped flap, with the longitudinal edges limited within the radial surface, and the distal edge made slightly exceeding the proximal interphalangeal joint line. (**c**) The volar incision. (**d**) The lateral view of the digit flap transfer, with direct suturing performed for closure of the donor site. (**e**) The volar view of the digit after flap transfer, with complete coverage of the volar skin defect.

A tongue-shaped flap was created on the radial wall of the 5th digit, with the longitudinal edges not exceeding the radial surface of the digit, and the distal edge made slightly beyond the PIP joint line. To ensure full coverage of the volar skin defect, the flap was made 2 mm larger in diameter than the recipient site (Fig. 2).

While creating the edges of the flap, care was taken to preserve the perforating blood vessels of the proper palmar digital arteries, as well as the proper palmar digital nerves.

Sequential release of affected soft tissues was performed in the following order—skin, subcutaneous fibrous fascia, flexor digitorum superficialis tendon, lumbrical muscle insertions if present, and volar plate. The degree of passive extension of the PIP joint was repeatedly tested, and surgical release was considered complete upon achieving full passive extension of the joint. Kirschner (K)-wire fixation was performed following volar plate release.

The radial flap was rotated 90° to cover the volar skin defect, and direct suturing was performed to close the donor site. Free skin grafting was indicated in the presence of high suture tension.

Mupirocin ointment and petroleum jelly (Vaseline) were subsequently applied, and the wound was wrapped with clean dressing. All digits were immobilized in the extended position with a cast for three weeks.

### Postoperative management

Following surgery, the affected limbs were elevated, and perfusion of the digits was closely monitored. Wound dressings, sutures, and K-wires were removed 2 weeks post-operation (3 weeks if volar plate release was performed). Thereafter, guided voluntary flexion and extension exercises were encouraged, in addition to an overnight splint to immobilize the digit in the extended position. Regular outpatient follow-ups were conducted, and treatment outcomes were assessed using the Mayo Clinic scoring system proposed by Siegert et al.^13^ (Table 2).

**Table 2:** The Mayo clinic scoring system proposed by Siegert et al.^6^ for treatment outcome.

Classification	Definition
Excellent	Correction to full extension, with < 15° loss of PIP joint flexion
Good	Correction to within 20° of full extension and > 40° increase in PIP joint extension, with < 30° loss of flexion
Fair	Correction to within 40° of full extension and > 20° increase in PIP joint extension, with < 45° loss of flexion
Poor	< 20° increase in PIP joint extension, with < 40° of total PIP joint motion

PIP, proximal interphalangeal.

## Data Availability

The datasets analyzed during the current study are available from the corresponding author on reasonable request.
